# Piercing the Shadows: Exploring the Influence of Signal Preprocessing on Interpreting Ultrasensitive Bioelectronic Sensor Data

**DOI:** 10.1002/cplu.202400520

**Published:** 2024-11-08

**Authors:** Mariapia Caputo, Lucia Sarcina, Cecilia Scandurra, Michele Catacchio, Matteo Piscitelli, Cinzia Di Franco, Paolo Bollella, Gaetano Scamarcio, Luisa Torsi, Eleonora Macchia

**Affiliations:** ^1^ Dipartimento di Farmacia-Scienze del Farmaco Università degli Studi di Bari “A. Moro” Via Edoardo Orabona 4 70125 Bari Italy; ^2^ Dipartimento di Chimica and Centre for Colloid and Surface Science Università degli Studi di Bari “A. Moro” Via Edoardo Orabona 4 70125 Bari Italy; ^3^ Dipartimento Interateneo di Fisica “M. Merlin” Università degli Studi di Bari “A. Moro” Via Edoardo Orabona 4 70125 Bari Italy; ^4^ CNR IFN Bari 70126 Italy; ^5^ The Faculty of Science and Engineering Åbo Akademi University Turku 20500 Finland

**Keywords:** Single molecule with a large transistor-SiMoT, Data preprocessing, Chemometric data processing, Pancreatic cancer early detection, Electrolyte gated field effect transistors

## Abstract

The development of ultrasensitive electronic sensors for in vitro diagnostics is essential for the reliable monitoring of asymptomatic individuals before illness proliferation or progression. These platforms are increasingly valued for their potential to enable timely diagnosis and swift prognosis of infectious or progressive diseases. Typically, the responses from these analytical tools are recorded as digital signals, with electronic data offering simpler processing compared to spectral and optical data. However, preprocessing electronic data from potentiometric biosensor arrays is still in its infancy compared to more established optical technologies. This study utilized the Single‐Molecule with a Large Transistor (SiMoT) array, which has achieved a Technology Readiness Level of 5, to explore the impact of data preprocessing on electronic biosensor outcomes. A dataset consisting of plasma and cyst fluid samples from 37 patients with pancreatic precursor cyst lesions was analyzed. The findings revealed that standard signal preprocessing can produce misleading conclusions due to artifacts introduced by mathematical transformations. The study offers strategies to mitigate these effects, ensuring that data interpretation remains accurate and reflective of the underlying biochemical information in the samples.

## Graphical abstract

The Single‐Molecule with a Large Transistor (SiMoT) array is used to explore the impact of data preprocessing on electronic biosensor outcomes. Cyst fluid and plasma samples from 37 patients with pancreatic precursor cyst lesions were analyzed. New strategies to mitigate these effects are provided, ensuring that data interpretation remains accurate and reflective of the underlying biochemical information in the samples.

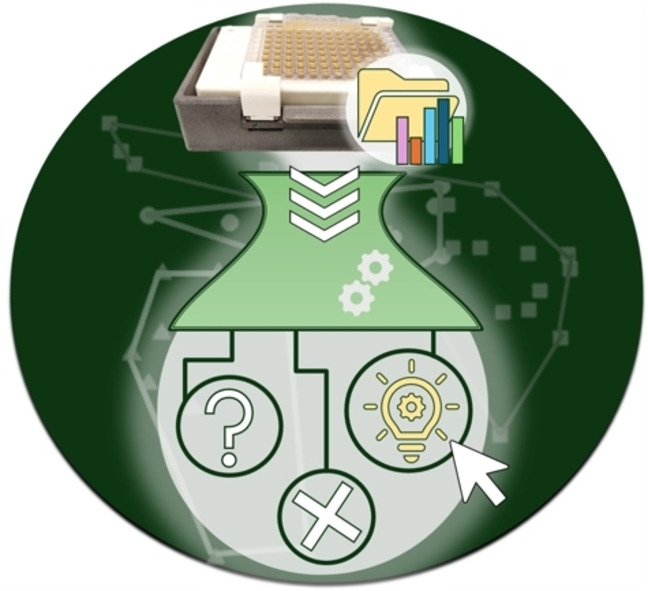

## Introduction

The detection of specific biomolecules, such as nucleic acids and proteins, alongside viruses and bacteria, is vital across a wide range of fields, including public health, environmental monitoring, food safety, agriculture, medicine, and veterinary science.[^1^, ^2^] Given the profound impact these biomolecules and pathogens have on food security, human health, and environmental sustainability, it is essential to rapidly develop innovative detection technologies capable of providing early and highly sensitive identification.[^3^, ^4^] Since Clark and Lyons introduced the first enzymatic biosensor in the early 1960s, there has been significant global research focused on biosensors.^[5]^ These analytical devices utilize biological recognition elements that specifically interact with target analytes, converting this interaction into a measurable signal *via* a transducer. The resulting signal is usually electrical, thermal, or optical. Notably, two distinct principles of signal transduction have been documented: *i)* label‐dependent technologies, which involve the analyte or recognition element conjugated with an optical or electroactive probe that is detected by the transducer, and *ii)* label‐free methods, which involve directly measuring a change in a physical variable generated during the recognition process. In this context, label‐free electronic biosensors offer significant advantages as they are engineered to act as key components in miniaturized, potentially fully integrated systems. These systems are able to detect target molecules and generating electronic responses, which are stored as digital signals represented by numerical matrices that reflect the intensities of the measured experimental variables. According to standard conventions, the rows of the dataset matrices represent vectors of analytical signals recorded from individual sampled solutions, while the columns represent the experimental variables measured. Given the multivariate nature of the collected data, analyzing just one variable or feature that varies with the target biomarker's concentration is highly risky and subjective, potentially leading to significant information loss. Therefore, the application of multivariate chemometric data processing is highly beneficial. However, these chemometric algorithms are rarely applied directly to the raw analytical data. Typically, appropriate mathematical preprocessing techniques are employed to reduce the impact of spurious signal variations, whether random or systematic.^[6]^ Given the inherent variability of complex biological samples like blood, serum, saliva, and urine, it is often not feasible to eliminate unwanted signal variations solely by adjusting instrumental parameters and experimental conditions. As a result, corrections are typically applied mathematically after the signal has been acquired and stored. These mathematical corrections are typically applied independently to the signal vectors from each individual sample, meaning each row of the data matrix is processed separately. Consequently, these techniques are commonly known as row pre‐processing. Many row preprocessing methods are available to address different spurious signals, including the minimization of random noise, drift (slope variations), baseline shifts (offsets), and global amplification effects, also known as size effects.[^7^, ^8^, ^9^, ^10^, ^11^, ^12^] Although row preprocessing offers many advantages for analyzing signals, these mathematical transformations can inadvertently change the data's structure. The effects of such changes on the results and conclusions are often greatly underestimated. Indeed, using improperly preprocessed signals can lead to a substantial risk of data overfitting, particularly when applying predictive modeling methods. Studies have shown that improper preprocessing of raw data can lead to incorrect interpretations of spectral features in both Near‐Infrared (NIR) and Mid‐Infrared (MIR) spectroscopy,[^6^, ^13^, ^14^] where features often coincide with poorly resolved overtones and combination bands. Although the misinterpretation of signals obtained from spectroscopic techniques has been thoroughly examined, these effects are critical not only for spectral data but may also affect a wide range of other instrumental signals. The Standard Normal Variate (SNV) transform, initially introduced by Barnes *et al*.,^[15]^ is one of the most commonly used row scaling techniques. Its primary objective is to ensure that all analytical signals are consistently represented.^[16]^ This means the signal intensities must be comparable and fall within a similar range across all samples in the dataset. Specifically, the SNV transform involves computing a scaling factor and applying it to each row (or sample) in the data matrix. Therefore, by implementing SNV row scaling, each sample becomes comparable to others by eliminating systematic biases, such as those arising from differences in sample collection, preparation, and analytical procedures.^[17]^ As a result, the SNV transform has become a preferred choice for various applications. It is extensively used not only for spectroscopic data, but also for signals of various other types, such as those obtained from potentiometric and amperometric sensors.[^18^, ^19^] However, the potential risks associated with applying such row preprocessing techniques to data collected from electronic sensors have been rarely investigated. In this study, we present an in‐depth investigation into the effects of row scaling on data collected using the ultrasensitive bioelectronic single‐molecule SiMoT array for the first time. The SiMoT technology encompasses two platforms that have achieved Technology Readiness Levels 5–6.^[2]^ On the first platform, a single biofunctionalized electrode (single‐sensor) is featured, designed to assay a single biomarker or pathogen, such as the SARS‐CoV‐2 virus.^[20]^ The second platform consists of a 96‐well microplate array, featuring biofunctionalized gate electric contact capable of simultaneously assaying multiple markers (multiplexing), which has been utilized for the timely screening of precursor cysts in pancreatic cancer. Remarkably, the SiMoT array has shown the capability to classify pancreatic cancer precursors through machine learning analysis, achieving a diagnostic sensitivity of at least 96 % and a diagnostic specificity of 100 %.[^21^, ^22^] This high performance is due to the array's capability to detect oncoproteins (MUC1 and CD55) and oncogenes (KRAS mutations, KRAS^mut^) at single‐molecule levels. In this investigation, data collected from a cohort of 26 unselected cyst fluid samples and 11 blood plasma samples were analyzed to assess the impact of row scaling on subsequent multivariate analysis. In particular, this study addresses the misinterpretation of results caused by row preprocessing of electronic signals, demonstrating how the SNV transform can lead to incongruous conclusions not supported by the underlying data. Additionally, we introduce a novel strategy to mitigate the unwanted effects of preprocessing, enabling a direct and unbiased interpretation of multivariate data analysis outcomes.

## Results and Discussion

### The SiMoT Array

**Figure 1:**
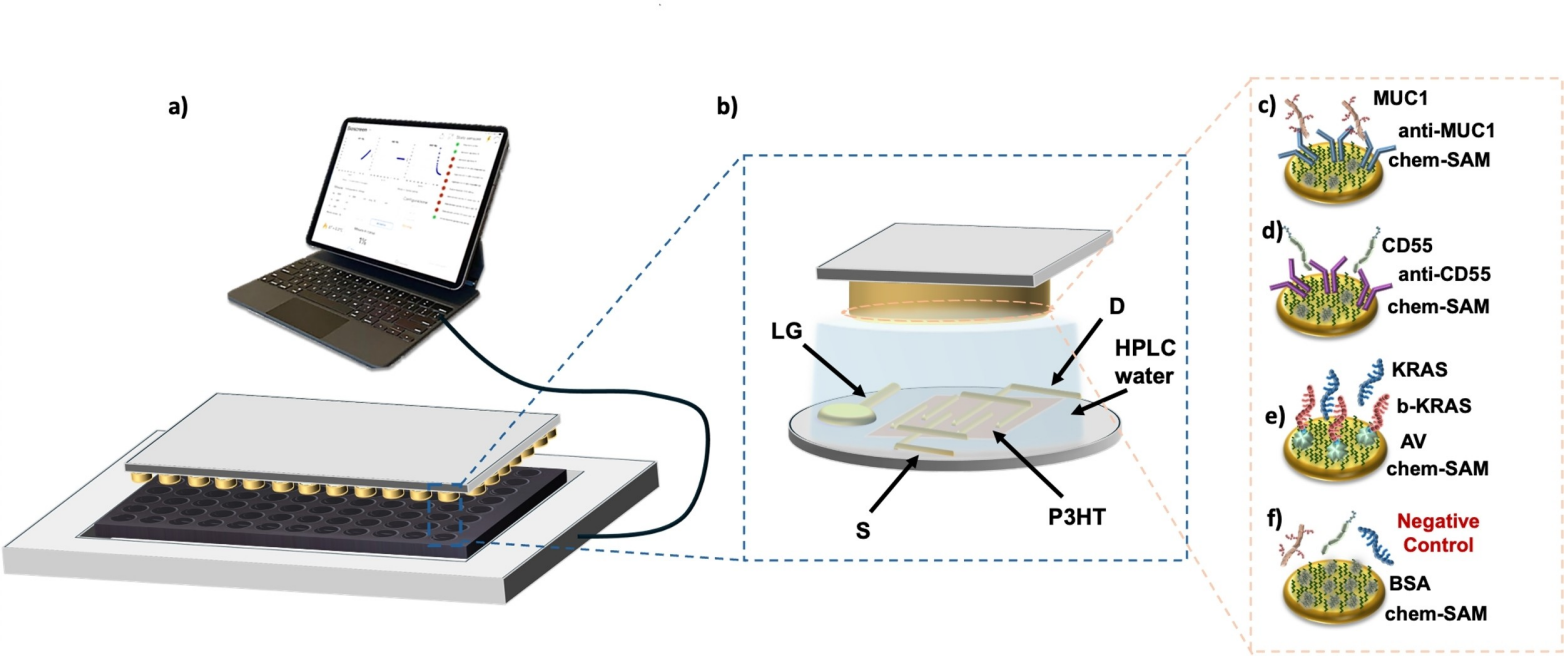
**(a)** Schematic representation of the SiMoT array, featuring the ELISA‐like disposable cartridge, the ELISA plate lid, displaying the sensing gates, and the case housing the read‐out electronics connected to a smart device. **(b)** Close‐up view of a single EG‐OFET device located at the bottom of each well in the 96‐well ELISA plate, along with a top biofunctionalized sensing gate. Visual depictions of biofunctionalized gates tested in triplicate, to the assay of **(c)** MUC1, **(d)** CD55 and **(e)** KRAS. **(f)** Schematic representation of the gate used for the negative control experiment, which is coated with Bovine Serum Albumin.

**Figure 2:**
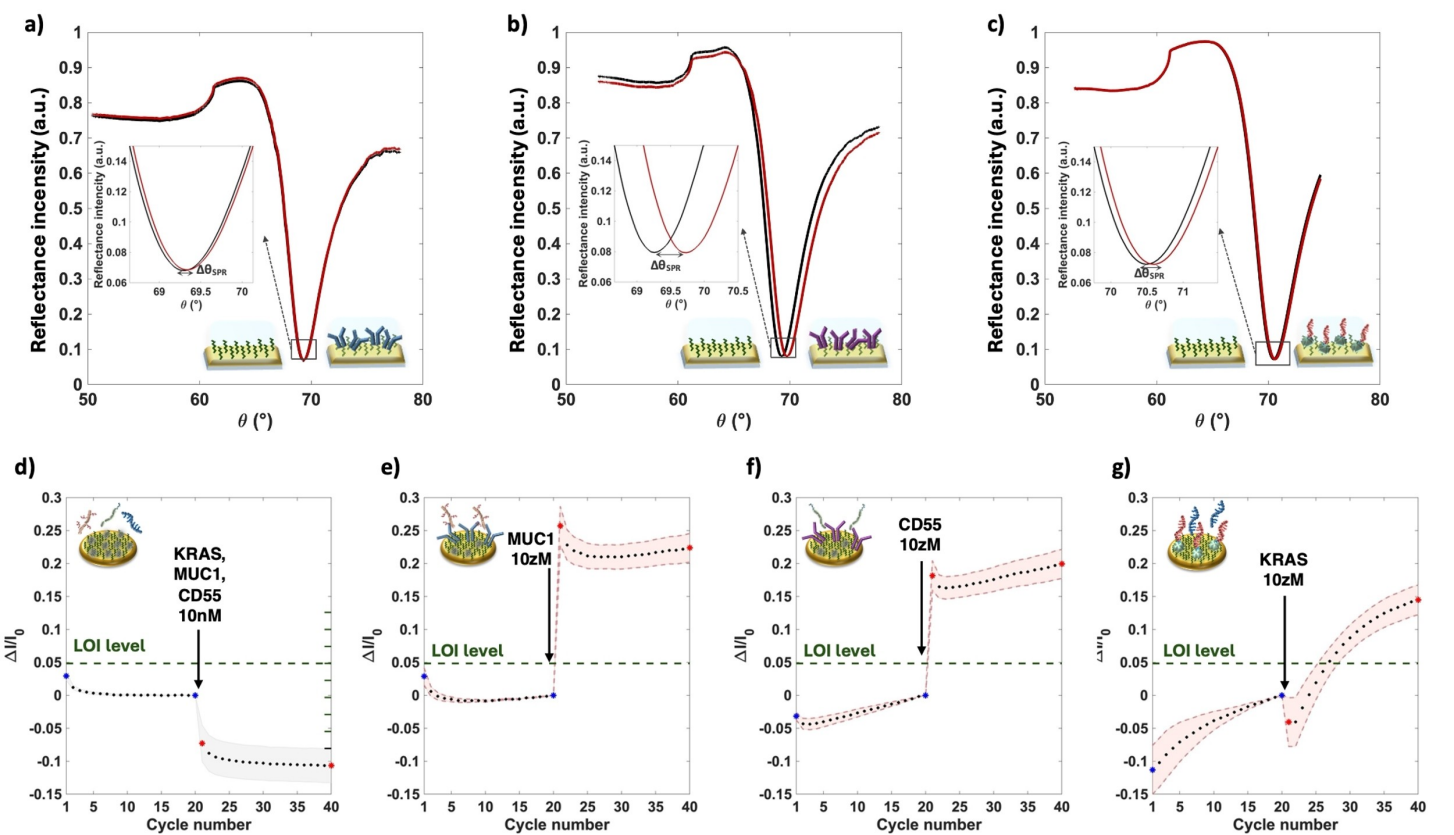
Plasmon peak reflectance intensity vs SPR angle measured for the deposition of **(a)** anti‐MUC1, **(b)** anti‐CD55 and **(c)** b‐KRAS. The black curve refers to the gold surface plus chemical SAM, while red curve refers to the modified surface with the three different bio‐recognition elements. In the inset, the angular shift measured after the deposition is shown. Current shift compared to the baseline current level measured when the gate is exposed to a concentration of 10 nM of **(d)** 7 negative control experiments, and to a concentration of 10 zM of **(e)** MUC1, **(f)** CD55 and **(g)** KRAS.

The SiMoT array, depicted in Figure 1, was designed to mimic the form factor of a conventional 96‐well ELISA plate, a staple tool in standard immunoassays. Specifically, it comprises a disposable cartridge designed to match the specifications of an 8×12 ELISA plate, as in Figure 1(a). The disposable cartridge is housed within a case that integrates the reusable reader. This reader includes a Silicon‐Integrated Circuit (Si‐IC) and a printed circuit board (PCB) module which can be connected to a standard smart device through a USB‐port. Moreover, each of the 96 wells houses an Electrolyte‐Gated Organic Field‐Effect Transistor (EG‐OFET), fabricated using cost‐effective additive manufacturing processes. As illustrated in Figure 1(b), each EG‐OFET features interdigitated source (S) and drain (D) electrodes, created by thermally evaporating gold onto a plastic substrate. Additionally, a coplanar Lateral Gate (LG) is incorporated to ensure the stability of the device. The semiconducting channel is formed by depositing a conjugated polymer, poly(3‐hexylthiophene) (P3HT), onto the interdigitated electrodes using ink‐jet printing. Deionized water dispensed into each ELISA well acts as the gating medium. Furthermore, Figure 1(b) illustrates a single top gate detecting interface of the SiMoT array. Indeed, the SiMot array is endowed with an ELISA plate lid, which features 96 gold‐coated pillars serving as the top sensing gates. Each of these gates is biofunctionalized by covalently attaching biorecognition elements to the gold surface, which ensures high selectivity for binding to specific markers. In particular, the sensing gate plate is divided into six areas, each containing 16 pillars dedicated to assaying a single patient's body fluid. Three replicates are conducted for each patient for all three biomarkers. For the detection process, capturing antibodies anti‐MUC1 and anti‐CD55 are covalently attached to the gold surface of the sensing gates, as shown in Figures 1c and d. For detecting KRAS the biofunctionalization process begins with avidin (AV) being covalently attached to the gold surface. This allows for an affinity binding with a biotinylated oligonucleotide strand (b‐KRAS), which subsequently hybridizes with KRAS^mut^, as depicted in Figure 1(e). Additionally, seven negative control experiments are conducted for each patient. These involve sensing gates coated with Bovine Serum Albumin (BSA), which are exposed to the patient's body fluid, as shown in Figure 1(f). The immobilization strategy for biorecognition elements, elaborated in detail elsewhere,^[23]^ achieves a surface density of approximately 10^12^ molecules per square centimeter segregated in the 25 mm^2^ sensing gate.^[22]^ The surface coverages obtained with anti‐MUC1, anti‐CD55, and b‐KRAS were assessed using Multi‐Parameter Surface Plasmon Resonance (MP‐SPR), and the resulting plasmons’ peaks are shown in Figure 2(a–c), respectively.^[24]^ The surface coverage of the adlayer of bio‐recognition elements anchored on the chemical SAM on the gold electrodes was determined through the angular shift (Δθ°) measured after the biorecognition elements deposition. The relation between the angle variation and the surface coverage expressed in ng/cm^2^ is defined by de Feijter's Equation^[25]^ Γ=d ⋅ (n‐n_0_) ⋅ (dn/dC)^−1^, where Γ (ng cm^−2^), is the surface coverage; d (nm) is the thickness of the biolayer deposited on the gold surface; (n‐n_0_) represents the difference between the refractive index of the layer and that of the bulk medium; and dn/dC is the so‐called “refractive index increment” of the adsorbed biolayer (with a value taken from literature, dn/dC≈0.182 cm^3^ g^−1^).^[26]^ Given the sensitivity factor of the instrument (k) at a laser wavelength of λ=670 nm, the refractive index variation can be expressed as (n‐n_0_)=Δθ_SPR_ ⋅ k. In this study for λ=670 nm, k is given as 86.3°/RIU (k=1.16 ⋅ 10^−2^ deg^−1^).[^27^, ^28^] Thus, de Feijter's Equation can be simplified as follows: Γ=d ⋅ Δθ_SPR_ ⋅ k ⋅ (dn/dC)^−1^. If a layer of bio‐recognition elements is deposited, considering the average dimension of an antibody and the AV‐b‐KRAS complex, which is within 10 nm (d~10^−6^ cm),[^29^, ^30^] the surface coverage can be approximated as Γ~64 ⋅ Δθ_SPR_. The surface coverage can be calculated thus for the three different biorecognition elements used (i. e. anti‐MUC1, anti‐CD55 and b‐KRAS). For anti‐MUC1 the angular shift is calculated as Δθ=(0.023±0.002)°, resulting in a surface coverage of Γ=(5.89±0.25) ⋅ 10^9^ anti‐MUC1 cm^−2^; for anti‐CD55 Δθ=(0.471±0.002)°, yielding a coverage of Γ=(1.21±0.01) ⋅ 10^11^ anti‐CD55 cm^−2^ and for b‐KRAS, Δθ=(0.115±0.002)°, with a corresponding surface coverage of Γ=(5.58±0.01) ⋅ 10^11^ b‐KRAS cm^−2^. The surface coverage is provided as the average of the data collected from four SPR traces inspecting two different slides. The variability in the surface coverage for the three biorecognition elements is within 5 %. Therefore a packing density of biorecognition elements in the range of 10^2^÷10^4^ molecules per square micrometer.^[31]^ This evidence confirms that a single biomarker within a 0.1 mL sample, diffusing according to Einstein's diffusion theory and acting as a Brownian particle, has a 95 % cross‐section probability of impinging on a biorecognition element segregated onto the sensing gate within a 10 minute incubation period.^[32]^

### Multiplexed Electronic Detection of Oncoproteins and Oncogenes

The SiMoT array sensing protocol comprises four main steps, enabling the concurrent analysis of samples from six patients. *(1)* Each well of a standard ELISA plate is filled with Phosphate Buffered Saline (PBS) reference fluid, and the sensing gate lid is incubated in this solution for 10 minutes. After incubation, the lid is washed with HPLC‐grade water. *(2)* The lid is placed onto the SiMoT cartridge, and the device is powered to record the baseline current level. This is achieved by running 20 consecutive transfer characteristic cycles, during which the drain current (I_D_) is measured while maintaining the drain voltage (V_D_) maintained at a constant of −0.4 V and varying the gate potential (V_GS_) is from 0 V to −0.5 V. The process is performed column‐wise, taking 2.5 minutes per column, resulting in a total of 30 minutes to record the baseline levels across the entire 96‐well SiMoT array. The I_0_ denotes the baseline current level. *(3)* The same sensing gate lid is exposed for 10 minutes to 0.1 mL of the assay solutions, such as blood plasma or cyst fluid, followed by washing. *(4)* The sensing gate lid is repositioned onto the SiMoT cartridge, and a new cycling measurement is conducted to define the sensing signal, denoted as *I*.

Furthermore, during the incubation steps *(1)* and *(3)*, the current level recorded by carrying the reference Lateral Gate (LG) is also monitored. If any transistor in the array shows a current shift greater than 10 % with the LG, its sensing signal is excluded from the analysis. The number of cycles was set at 20 for the acquisition of transfer characteristics to ensure the recording of a stable baseline and sensing signal while minimizing the assay time‐to‐result. Increasing the number of cycles would have significantly impacted the time‐to‐result without substantially improving the quality of the extracted electronic features.

Recent studies have demonstrated that the SiMoT array functions as an *on/off* sensor by setting the decision threshold at the Limit of Identification (LOI).[^20^, ^33^] The LOI represents the minimum detectable quantity of a particular analyte that can be reliably distinguished from statistical noise, with a confidence level greater than 99 %, thus maintaining false‐negative and false‐positive rates below 1 %.^[2]^ This threshold is established at the average noise level, defined as the mean signal of the negative control experiments (μ) plus six times the standard deviation (6 σ), as detailed in previous research.[^34^, ^35^] Remarkably, a significant shift in current *I* compared to the baseline current level *I_0_*, surpassing the LOI threshold, is detected when the sensing gate is exposed to a target biomarker concentration as low as a 10 zM, whether it be an oncoprotein (MUC1 or CD55) or a mutated oncogene (KRAS), as shown in Figure 2(d–g).[^22^, ^33^] This indicates that even 1±1 molecule of the analyte in a 0.1 mL sample can trigger a biochemical amplification effect, currently under investigation, altering the work function of the entire sensing gate.[^36^, ^37^] Blood plasma and cyst fluid from a cohort of 37 patients were analyzed using the SiMoT array sensing protocol, as detailed in Table 1, which summarizes the data matrix. Each row in the matrix represents a different patient sample, while the first two columns categorize the type of body fluid analyzed, either plasma (P) or cyst fluid (C), and the diagnosis based on current diagnostic standards. The latter was assessed through conventional diagnostic methods, including demographic data, Next‐Generation Sequencing (NGS) and histo‐cytological analysis. Specifically, patients were classified into potentially non‐mucinous cysts (N), which include benign and non‐mucinous cysts or pseudocysts as a control group, and potentially low‐grade (LG) and high‐grade (HG) mucinous cysts, as indicated in Table 1. Identifying high‐grade mucinous lesions is crucial due to their high risk of transforming into invasive cancer, necessitating immediate surgical intervention. In contrast, patients with potentially non‐mucinous cysts can be discharged, and those with potentially low‐grade lesions can enter a follow‐up protocol. For each sample, the oncoproteins CD55 and MUC1, and the mutated oncogene KRAS^mut^ were tested using the SiMoT assay in triplicate.

**Table 1:** SiMoT array data matrix, where each row reports the analytical signals collected for each patient. The column report two categorical variables, namely the type of fluid assayed, either plasma (P) or cyst fluid (C), and the state‐of‐the‐art diagnosis. The other 9 columns are relevant to the SiMoT features f_1_, f_2_, and f_3_ extracted for each biomarker. Each feature is reported as the average over three replicates.

			MUC1			KRAS			CD55		
ID	Fluid	Class	f_1_	f_2_	f_3_	f_1_	f_2_	f_3_	f_1_	f_2_	f_3_
S1	P	HG	0.22	1.65	1	0.14	2.67	1	0.14	0.48	1
S2	P	HG	0.60	1.50	1	0.61	0.98	1	0.27	0.57	1
S3	P	HG	0.13	0.68	1	0.17	0.81	1	0.54	0.25	1
S4	C	HG	0.25	0.62	1	0.16	0.86	1	0.18	0.82	1
S5	C	HG	0.14	4.55	1	0.20	2.00	1	0.57	0.35	1
S6	C	HG	0.69	0.68	1	0.27	1.11	0	0.39	0.04	1
S7	C	HG	0.14	1.74	1	0.03	5.00	1	0.13	0.50	1
S8	C	HG	0.19	2.82	1	0.00	0.00	0	0.09	0.17	1
S9	C	HG	0.86	1.80	1	0.20	1.00	1	0.27	1.11	1
S10	P	LG	0.07	0.81	1	−0.02	0.22	0	−0.03	2.25	0
S11	P	LG	0.37	0.51	1	0.09	−1.00	0	0.11	−0.70	0
S12	P	LG	0.18	1.09	1	0.01	−0.78	0	0.04	−6.67	0
S13	P	LG	0.08	0.63	1	−0.26	−1.39	0	−0.29	−0.64	0
S14	P	LG	0.19	1.69	1	−0.02	0.07	0	−0.12	−0.29	0
S15	C	LG	0.09	0.33	1	−0.07	0.00	0	−0.07	−0.96	0
S16	C	LG	0.48	1.40	1	0.34	−2.00	0	0.32	−1.91	0
S17	C	LG	0.19	0.52	1	0.04	−0.59	0	0.01	−0.54	0
S18	C	LG	0.07	1.40	1	0.05	−1.00	0	−0.02	−1.05	0
S19	P	LG	0.16	0.95	0	−0.30	−0.69	0	−0.30	−0.57	0
S20	P	N	−0.37	−0.41	0	−0.32	−0.67	0	−0.26	−0.63	0
S21	P	N	−0.31	−1.26	0	−0.31	−0.65	0	−0.28	−3.77	0
S22	C	N	0.14	1.31	0	0.04	−0.07	0	0.05	−0.46	0
S23	C	N	−0.11	−0.43	0	−0.09	−0.31	0	−0.02	−0.48	0
S24	C	N	0.65	−0.38	0	0.00	0.00	0	−0.02	−1.00	0
S25	C	N	−0.20	−0.80	0	−0.16	−0.67	0	−0.12	−2.00	0
S26	C	N	−0.14	−2.38	0	−0.11	−3.24	0	−0.09	−2.23	0
S27	C	N	−0.04	−0.92	0	−0.04	−0.60	0	−0.07	−0.25	0
S28	C	N	0.24	0.58	0	−0.13	−0.50	0	0.34	−7.00	0
S29	C	N	0.12	−1.15	0	0.03	−1.25	0	0.07	−2.00	0
S30	C	N	0.18	0.91	0	−0.17	−0.50	0	0.00	0.00	0
S31	C	N	−0.02	−2.26	0	0.23	−1.11	0	0.09	−0.26	0
S32	C	N	−0.02	0.00	0	−0.15	−1.14	0	−0.10	−0.50	0
S33	C	N	0.12	1.11	0	0.01	0.14	0	0.03	1.00	0
S34	C	N	0.13	2.90	0	0.11	1.00	0	0.14	0.60	0
S35	C	N	0.00	0.00	0	−0.01	−0.68	0	0.00	0.00	0
S36	C	N	0.15	0.52	0	0.19	0.67	0	0.21	1.00	0
S37	C	N	0.24	0.39	0	0.08	0.73	0	0.07	−1.94	0

**Figure 3:**
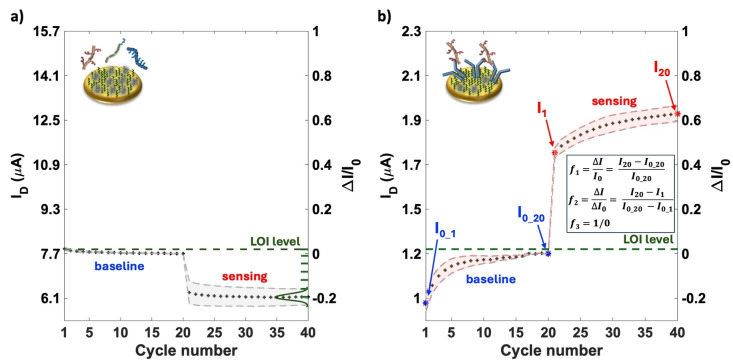
Representative example of the I_D_ current data points measured at V_D_=−0.4 V and V_G_=−0.5 V vs. cycles in a well of a SiMoT multiplexing array. **(a)** Average current data points along with the standard deviation (grey shadow) measured for 7 negative control experiments (see main text for details) with BSA biofunctionalized gates. These experiments provided the Gaussian distribution of the noise's statistical parameters and the LOI level. **(b)** Data‐points for a MUC1 assay in a cyst fluid are given as the average of three replicates on three distinct gates while the shaded area are the standard deviations. Operatively, the gate functionalized with anti‐MUC1is at first incubated in PBS and the baseline (I_0_1–20,_ blue labels) is measured in deionized water; subsequently the same gate is incubated in plasma sample from a high‐grade mucinous cyst patient and the sensing current (I_1–20_, red labels) is measured in water. The dashed green line is the LOI level (see panel a) while f_1_, f_2_, and f_3_ are the features used for the Principal Component Analysis (see text for details).

It is worth mentioning that the Gaussian distribution of the noise's statistical parameters, namely the LOI level, was evaluated through the seven negative control experiments for each patient, encompassing BSA biofunctionalized gates exposed to the patient's body fluid. Figure 3(a) illustrates typical data points of the I_D_ current measured at V_D_=−0.4 V and V_GS_=−0.5 V across the cycle numbers with the seven BSA‐coated gates, that is the negative control experiments. The graph includes the average results from the seven negative control experiments, with the standard deviation represented by a grey shadow. The initial 20 cycles were recorded after a 10 minute incubation of the biofunctionalized gate in PBS. The subsequent 20 cycles were measured after a 10 minute incubation of the same gate in the patient's fluid. The Gaussian distribution of the noise is shown in Figure 3(a) as a dark green line, while the dashed dark green line corresponds to the LOI level. These measurements were conducted in deionized water (HPLC grade) to minimize the screening of electrostatic effects and maximize the Debye length. In Figure 3(b), the data for a typical assay of the MUC1 oncoprotein in a patient's plasma sample diagnosed with a high‐grade mucinous cyst is presented. The data points represent the average current levels, *I_0_* (from cycles 1 to 20) and *I* (from cycles 21 to 40), for three replicates. The relative standard deviation is indicated by the shaded area. Three features, *f_1_*, *f_2_*, and *f_3_*, are extracted from this sensing data. Feature *f_1_* represents the relative change of the signal compared to the baseline, namely the $\Delta I/I_{0}$ (see Experimental Section). Feature *f_2_* captures the slope of the current drift throughout the cycling process, specifically, the ratio between the current drift observed during the sensing cycles and that recorded during the baseline phase. Feature *f_3_* evaluates the *f_1_* of each marker against the patient's LOI levels. It assigns a value of 0 if *f_1_* is below the LOI (indicated by the dashed dark green line in Figure 3) and a value of 1 if *f_1_* is above the LOI. These three features are considered the key elements of each sensing assay and correspond to the nine variables reported in the data matrix in Table 1 for each biomarker. Each feature in Table 1 represents the average of the three replicates, measured using three different gates of the array, each exposed to different aliquots of the patient's fluid. The percentage error among the three replicates is within 5 % for *f_1_* and reaches a maximum of 15 % for *f_2_*.

### Assessment of Normalization Performance

**Figure 4:**
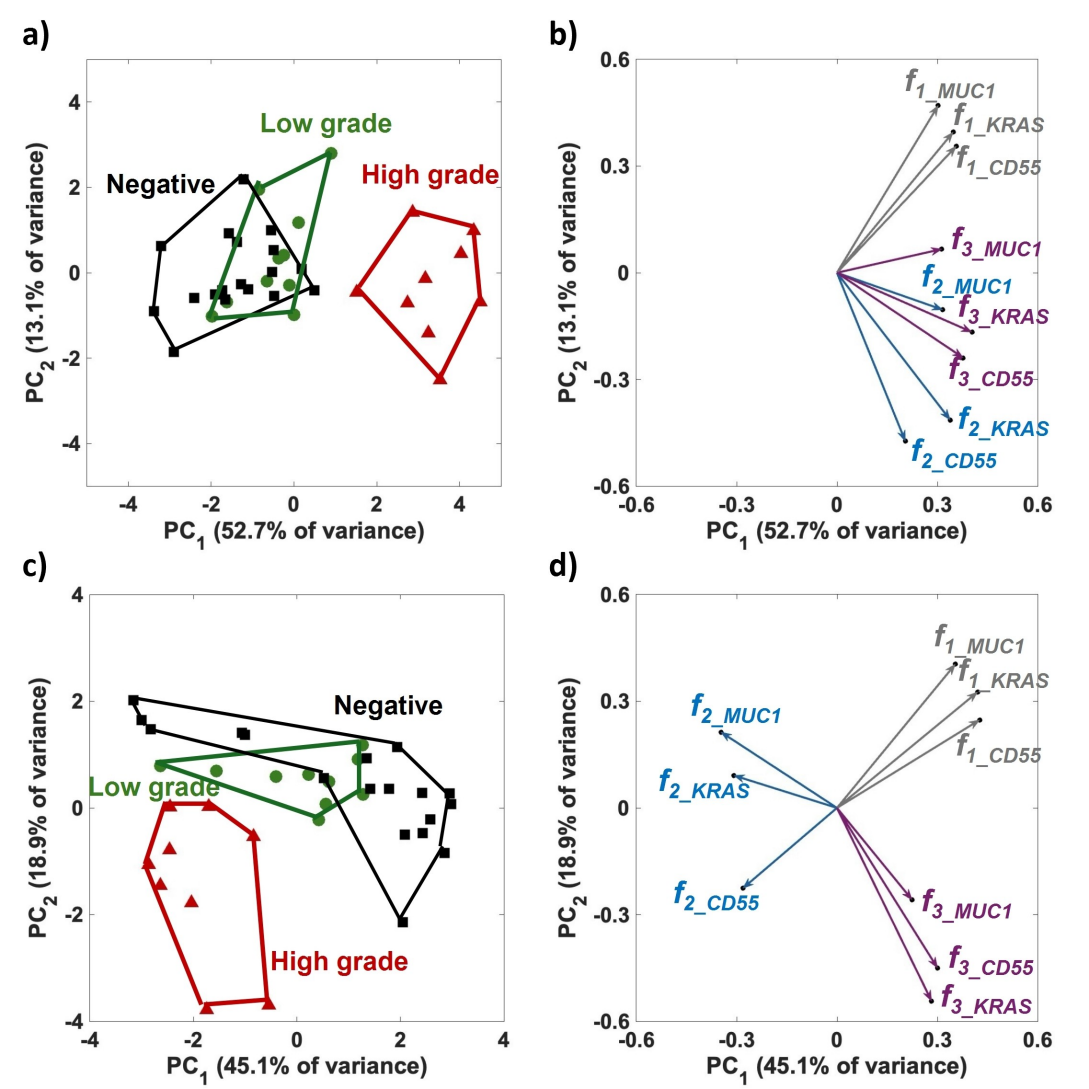
**(a)** Score plot evaluated on the SiMoT array data matrix after column autoscaling. Red, green, and black labels represent the high‐grade, potentially low‐grade, and potentially non‐mucinous cysts, respectively. PC1 explains the 52.7 % of the variance, while PC2 the 13.1 %. **(b)** Loading scatter plot, depicting how each feature contributes to PC1 and PC2; the features f1, f2, and f3 are represented by grey, blue, and violet arrows, respectively. **(c)** Score‐Plot and **(d)** Loading scatter plot evaluated on the SiMoT array data matrix after SNV row scaling. PC1 explains the 45.1 % of the variance, while PC2 the 18.9 %.

An investigative multivariate data analysis was conducted on the dataset presented in Table 1, employing Principal Component Analysis (PCA). PCA is a widely used chemometric method for visualizing latent data structures through graphical plots, offering deeper insights than analyzing individual variables alone.^[38]^ By considering all variables simultaneously, PCA facilitates comprehensive interpretations. Typically, PCA is the initial step in analyzing a multivariate dataset, though further analysis with more advanced methods may also be necessary. PCA is particularly useful for data characterized by non‐trivial correlations among some, most, or all variables involved. For each of the three markers, PCA provides different principal components (PCs) that are orthogonal to each other. These components are expressed as linear combinations of the features *f_1_*, *f_2_*, and *f_3_*, with weights determined by the eigenvalues of the *loading matrix*. The values of these new variables in the PC space are represented by the *scores*. The primary aim of the PCA analysis was to assess the effectiveness of different preprocessing strategies and their impact on data interpretation. Figure 4 (a) and (b) present the results of the exploratory PCA conducted on the dataset in Table 1, following the customary column autoscaling procedure.^[39]^ Column autoscaling, as detailed in the Experimental Section, standardizes each feature so that its mean is zero and its standard deviation is one. This step is essential because, without it, features with larger values would dominate the analysis. Autoscaling ensures that each variable contributes equally to the PCA, although the relative distribution of data points in the high‐dimensional measurement space is not altered. The PCA analysis reveals that samples can be well characterized based on their diagnosis, revealing distinct clusters in the orthogonal space defined by PC1 and PC2. Figure 4(a) displays the *scores* on PC1 and PC2. It is evident that the high‐grade samples (red triangles) form a well‐defined cluster, while potentially non‐mucinous cysts (black squares) and potentially low‐grade (green circles) samples exhibit significant overlap. The high‐grade samples are clustered at positive PC1 *scores*, whereas the potentially low‐grade and non‐mucinous cysts are located at negative PC1 values. Figure 4(b) shows the *loadings* on PC1 and PC2. They indicate the weights assigned to features *f_1_*, *f_2_*, and *f_3_*. Loadings can vary between −1 to 1, with values close to either −1 or 1 suggesting a strong influence of that feature on the principal component.^[40]^ Conversely, loadings close to zero suggest that the feature has a minimal impact on the principal component. The analysis indicates that the presence of any feature for all three markers is characterized by positive loadings on PC1. Given that the features analyzed in this study are all correlated with the presence of oncoproteins and mutated oncogenes, it is expected that the loadings predominantly show positive values on PC1.

Moreover, the two oncoproteins are partially distinguished by opposite PC2 loadings: MUC1 shows very positive loadings while CD55 is characterized mostly by negative loadings. The *loadings* interpretation suggests that samples with high *score* values for a given PC are positively characterized by variables with high loading values for the same PC.^[6]^ Consequently, the high‐grade samples, which all exhibit positive scores on PC1, are characterized by the presence of all three markers. In contrast, non‐mucinous cysts, where the three markers are not detected, are positioned at negative PC1 values. Furthermore, an overlap is observed between potentially low‐grade cysts, characterized by the presence of only the MUC1 oncoprotein, and potentially non‐mucinous cysts.

It is important to note that row preprocessing of variables can impact the shape of the loadings.^[40]^ Furthermore, to ensure that the observed loading structure is not due to unwanted bias due to the different biochemical compositions of the analyzed samples, the Standard Normal Variate (SNV) transformation was applied to the data matrix, before performing the PCA analysis in Figure 4(c) and (d). Figures 4(c) and (d) display the score plot and loading profile after SNV row scaling.

The application of SNV normalization to the SiMoT dataset significantly reduces the overlap between potentially non‐mucinous and potentially low‐grade cyst classes, thereby enhancing the distinction within the PC1 *vs*. PC2 score space, as shown in Figure 4(c). However, analyzing both the *score* and *loading* plots together shows that the high‐grade samples are characterized by negative score values on PC1, indicating a negative association with features *f_1_* and *f_3_*. This leads to an incongruous conclusion, suggesting that high‐grade samples are characterized by the absence of all three markers, a scenario typically referred to as the loading paradox, as shown in Figure 4(d). While the SNV transformation improves clustering quality in the confusion area of the score plot, it does so at the cost of losing crucial information related to the original variables. Consequently, the SNV transformation introduces artifacts that distort the fundamental biochemical information underlying the SiMoT assay.

This demonstrates that the selection of a normalization method significantly influences the subsequent steps in multivariate analysis for electronic signals. Choosing an appropriate method that aligns with the data characteristics can be challenging and demands a thorough understanding of both the instrumentation used and the biological system under investigation. To this end, performing PCA on the raw data can offer valuable insights into selecting an appropriate normalization method. Additionally, minimizing the impact of noise, which can significantly contribute to spurious correlations, is a highly effective strategy for preventing the introduction of artifacts resulting from mathematical transformations. Noise is inherently present in all datasets and can arise from instrument‐related interferences while collecting data or from the natural yet stochastic variation of the biological system under study. Typically, noise is categorized into two types. *i)* Homoscedastic noise, which is independent of the measured values and associated to the lowest detection limit, and *ii)* heteroscedastic noise, which correlates with the measured analytical signals and increases as the measured signal increases. Data affected by heteroscedastic noise often leads to a significant number of false correlations, whereas these correlations are less pronounced when the noise is homoscedastic. Therefore, strategies should be employed to convert heteroscedastic noise into homoscedastic one as much as possible.

To address this, noise levels were assessed for each patient's fluid sample using the SiMoT array, which effectively minimizes unwanted biases from spurious signals during the analysis of real biofluids. The complex nature of these biofluids can introduce variations in signals across multiple arrays, potentially masking the true expression of oncoproteins or oncogenes. Therefore, defining the decision threshold based on the noise level (LOI) evaluated for each patient is crucial for preventing heteroscedastic noise. This approach contrasts with defining the LOI based on biofluids from different patients, which would result in higher heteroscedastic noise component. By introducing a noise level specific to each sampled fluid, we effectively convert heteroscedastic noise into homoscedastic one, thereby significantly reducing spurious correlations.

Additionally, another source of variability stems from the use of an array of devices. For the SiMoT array, this involves an intrinsic device‐to‐device variation in the electrical transfer characteristics registered with the LG electrode of approximately ±60 %.^[33]^ The biofunctionalization protocol used in fabricating the sensing gate introduces further array bias due to gate‐to‐gate variations in the dielectric, morphological, and mechanical properties of the deposited biolayer. Although recent findings indicate that applying an electric field to a biofunctionalized gate can reduce the dispersion, a variation of about 20 % in the electronic signals collected from different sensing gates still persists.^[41]^ Therefore, the most effective normalization strategy to address these array biases involves defining features based on comparing the signal *I* registered upon exposure to the patient sample with the baseline *I_0_* level, which is evaluated on the very same well of the SiMoT device. The proposed methodology enables direct and unbiased interpretation of the sensing data collected with the SiMoT array platform. This approach has been proven to yield more interpretable and robust loadings after applying the PCA model, compared to standard row‐scaling techniques.

## Conclusions

The present study examines the impact of standard row‐preprocessing tools on interpreting data collected with potentiometric sensors. These tools typically represent a preliminary step in the multivariate analysis of analytical signals. While the drawbacks of applying the SNV transform to spectroscopic data have been extensively investigated, its impact on electronic analytical signals collected with sensor arrays remains largely unexplored. This study demonstrates that such a widespread mathematical transformation may introduce artifacts, distorting the biochemical information within the data matrix.

To address these challenges, a new strategy has been introduced, that allows for a straightforward interpretation of the significance of the original features and ensuring a reliable explanation of the biochemical information characterizing the pancreatic precursor cyst's samples. This was achieved by applying PCA to the SiMoT array dataset, focusing on three features (*f_1_*, *f_2_*, and *f_3_*) defined for each biomarker. These features were selected to minimize device‐to‐device fluctuations and control the biochemical noise level. The features *f_1_* and *f_2_*, encompassing a normalization for the baseline current level *I_0_* of the specific device addressed in each sensing experiment, mitigate the effect of the array biases. On the other hand, the noise was minimized by defining the LOI for each assayed patient's biofluid and incorporating this threshold into the *f_3_* feature. A combined interpretation of *scores* and *loadings* allowed for efficient information extraction, correlating different clusters of potentially non‐mucinous, potentially low‐grade, and high‐grade cysts and to the original variables.

This approach is generalizable and widely applicable to various new analytical array technologies, which are gaining momentum for ultrasensitive clinical applications.

## Experimental

*Fabrication of the SiMoT array*: A flexible PEN foil was used to fabricate the array, with a lift‐off photolithography technique. At first, a 2 nm layer of chromium was deposited as an adhesion layer, and then 30 nm of gold was evaporated onto it. The interdigitated source and drain electrode configuration, designed after cleaning the substrates, included a coplanar gate with a diameter of 2.5 mm, and a channel width and length of 10^4^ μm and 5 μm, respectively. The gold connectors were covered with an SU8 inkjet‐printed film, leaving the coplanar gate areas and the electronic channel uncovered. Afterwards, the gold drain and source contacts were coated with the organic semiconductor P3HT.

*Fabrication of 3D Cover Plates*: The SiMoT sensing gate plates were produced using 3D stereolithography printing. After printing, the samples were heated in a UV oven at a temperature of 65 °C for 20 minutes, followed by a 30 minute exposure to UV light. To reduce surface roughness before gold evaporation, a Parylene‐C layer of 2 μm was applied through Chemical‐Vapor‐Deposition (CVD). A 150 nm thick gold layer was then applied via electron‐beam evaporation. Post‐deposition, the samples were cleaned using solvents of increasing polarity and ozone treatment before proceeding with biofunctionalization.

*Biofunctionalization of 3D Sensing Gates*: Initially, a chemically self‐assembled monolayer was immobilized on the sensing gates’ surface. For this purpose, a solution of 10 mM 3‐mercaptopropionic acid and 11‐mercaptoundecanoic acid (molar ratio of 10 : 1), was prepared in ethanol. The gates were then treated with a 200 mM solution of 1‐Ethyl‐3‐(3‐dimethylaminopropyl) carbodiimide and 50 mM sulfo‐N‐hydroxysuccinimide in water for 20 minutes at a temperature of 21 °C. The steps used in the biofunctionalization process are as follows: *1)* The sensing interface was incubated in PBS solutions containing antibodies, i. e. anti‐CD55 or anti‐MuC1, for 2 hours at a temperature of 21 °C. This was followed by treatment with 1 M ethanolamine in PBS at 21 °C for 45 minutes. The biofunctionalized gate was then exposed to a BSA solution in PBS for 1 hour at 21 °C.

*2)* Alternatively, the gate was incubated in a solution containing AV in PBS for 2 hours at 21 °C. The AV‐modified self‐assembled monolayer was then exposed to 1 M ethanolamine in PBS at a temperature of 21 °C for 45 minutes, followed by immersion in a 0.5 μM b‐KRAS PBS solution for 1 hour at 21 °C. Negative control experiments involved only the covalent bonding of BSA to the chemical SAM.

*Body Fluid Collection*: Sample fluids were collected at the Düsseldorf Institute of Pathology, with storage at −80 °C. To remove residual cells, pancreatic cyst fluids were centrifuged at 1600 x g for 10 minutes at 21 °C, and then were diluted at a 1 : 8 (v/v) ratio in PBS. Human blood plasma was centrifuged at 10,000 x g for 5 minutes at 21 °C, then diluted at a 1 : 8 (v/v) ratio in PBS. Before the analysis with gates biofunctionalized with b‐KRAS, diluted samples from cyst fluids or plasma were heated at 90 °C for 3 minutes. The study has received ethic approval by the Ethic Commission of the University Hospital of Düsseldorf concerning analysis of body fluids and tissues.

*SiMoT Array Sensing Protocol: The SiMoT assays were operated using a non‐regenerative approach*. The 8×12 SiMoT array was immersed in HPLC‐grade water for approximately 24 hours to ensure the stability of the source‐drain current (*I_D_*). The I_D_ vs. V_GS_ values of the lateral gates were recorded at a constant V_D_ every 30 minutes, continuing until the current drift was reduced to below 5 % per day. The sensing gates were first incubated in PBS (0.1 mL per well) for 10 minutes, followed by a thorough rinse with HPLC water. Cycling measurements over 2.5 minutes were conducted to establish a stable baseline (*I_0_*). Patient specimens were exposed to the same gate plate for 10 minutes, followed by washing with HPLC water. After that new cycling measurements were acquired. The stability of the device was monitored during both incubation phases by measuring the *I_D_* traces using the lateral gate. Data analysis was performed using MATLAB software.

*The SiMoT feature extraction*: The analysis of CD55, MUC1, and KRAS^mut^ for each patient fluid was conducted simultaneously. Additionally, seven negative control experiments were analysed to determine the LOI level. The LOI level represents the minimum concentration of the analyte that can be reliably differentiated from the noise level with a confidence level better than 99 %. This ensures that the occurrence of false‐positive and false‐negative is less than 1 %. LOI level is set at the mean of the noise (μ_*n*_) plus 6 times its standard deviation (6 σ). Table 1 provides a summary of the measurements in addition to the final diagnosis and the biofluid used for SiMoT analysis. The features were calculated in the same way for each biomarkers as follows: *i) **f***_***1***_=*(I_20_−I*_*0_20*_ *)/I*_*0_20*_ (average over three replicates). *ii) **f***_***2***_=$\frac{I_{20}-I_{1}}{I_{0\_20}-I_{0\_1}}$ , where *I*_*1,*_ *I_20_*, *I*_*0_1*_ *I*_*0_20*_ are the *I* and *I_0_* current values (at V_D_=−0.4 V and V_GS_=−0.5 V) measured at cycles 1 and at cycle 20. This feature corresponds to the normalized dynamic drift of a specific gate. *iii) **f***_***3***_ is a binary variable (0, 1) where 0 indicates that the ΔI/I_0_≤LOI level, and 1 indicates that ΔI/I_0_>LOI.

*SiMoT data pre‐processing*: All analyses were performed using the open‐source software CAT (Chemo‐metric Agile Tool).^[42]^ The first data pre‐processing method used was the *column autoscaling*. Autoscaling involves two main steps. The first step is the *mean centering*, achieved by subtracting the mean value of each column from each value of the input matrix. The second step is *scaling*, in which the data values are divided by the standard deviation of each column, resulting in variables with unit variance.

The transformed values after column autoscaling is given by $x_{ij}^{Autoscaled}=\frac{x_{ij}-\mu_{j}}{\sigma_{j}}$ , where *x_ij_* is the value of the *j* ^*th*^ varible of the *i* ^*th*^ sample, while μ_j_ and σ_j_ are the mean and the standard deviation of each j^th^ variable column‐wise, respectively.

Autoscaled data have zero mean and unit variance, thereby giving all variables an equal statistical weight. The second data pre‐processing method applied is the *Standard Normal Variate* (SNV). This method normalizes each row of the input matrix by subtracting the mean of the row and then dividing by its standard deviation. Given a generical matrix *X_ij_* the SNV‐transformed value *x_ij_* ^*SNV*^ is given by $x_{ij}^{SNV}=\frac{x_{ij}-\mu_{i}}{\sigma_{i}}$ , where *x_ij_* is the value of the *j* ^*th*^ varible of the *i* ^*th*^ sample, while μ_i_ and σ_i_ are the mean and the standard deviation of all the variables of each i^th^ sample row‐wise, respectively.

## Conflict of Interests

The authors declare no conflict of interest.

1

## Data Availability

The data that supports the findings of this study are available within the article.
